# A new perspective of vasculogenic mimicry: EMT and cancer stem cells (Review)

**DOI:** 10.3892/ol.2013.1555

**Published:** 2013-08-30

**Authors:** YUN-LONG FAN, MIN ZHENG, YA-LING TANG, XIN-HUA LIANG

**Affiliations:** 1 State Key Laboratory of Oral Diseases, West China Hospital of Stomatology, Sichuan University, Chengdu, Sichuan 610041, P.R. China; 2 Department of Oral Pathology, West China Hospital of Stomatology, Sichuan University, Chengdu, Sichuan 610041, P.R. China; 3 Department of Oral and Maxillofacial Surgery, West China Hospital of Stomatology, Sichuan University, Chengdu, Sichuan 610041, P.R. China

**Keywords:** cancer stem cell, vasculogenic mimicry, epithelial-mesenchymal transition, angiogenesis, metastasis

## Abstract

Vasculogenic mimicry (VM), a new pattern of tumor microcirculation, is important for the growth and progression of tumors. Epithelial-mesenchymal transition (EMT) is pivotal in malignant tumor progression and VM formation. With increasing knowledge of cancer stem cell (CSC) phenotypes and functions, increasing evidence suggests that CSCs are involved in VM formation. Recent studies have indicated that EMT is relevant to the acquisition and maintenance of stem cell-like characteristics. Thus, in this review we discuss the correlation between CSCs, EMT and VM formation.

## 1. Introduction

For many years, angiogenesis via the sprouting of new vessels from existing ones was considered to be the exclusive method of tumor vascularization (1), and anti-angiogenesis therapies were applied as a promising method to ‘starve’ tumors. However, with the administration of angiogenesis inhibitors primarily targeting endothelial cells, it was identified that the effect of these types of drugs was limited. This indicated that there may be other supplementary blood supply patterns used to nourish tumors. In 1999, Maniotis *et al*(2) first reported that highly aggressive and metastatic melanoma cells are able to form highly patterned vascular channels lined externally by tumor cells, without the existence of endothelial cells. This process was termed vasculogenic mimicry (VM), which is independent of angiogenesis, and is composed of tumor cells and a basement membrane. VM was categorized into two distinctive types: the patterned matrix type (2) and the tubular type (3). Blood plasma and red blood cells are able to flow in the nonendothelial cell-lined vessel-like structures (2,4), and a VM-angiogenesis junction in the central area of the inflammatory breast cancer (IBC) xenografts has been observed (5). This evidence suggests that VM in the tumor mass is connected with host vessels for blood supply and is part of the functional microcirculation.

Subsequently, VM has been observed in numerous types of aggressive tumors, such as colorectal cancer (6), head and neck squamous cell carcinoma (HNSCC) (7–9), glioblastoma (3,10), breast cancer (11,12), ovarian carcinoma (13,14), astrocytoma (15) and Ewing sarcoma (4). Increasing evidence has suggested that patients with tumors undergoing VM have a worse prognosis and VM may be used as an independent predictor of prognosis (16–18). Lin *et al*(9) analyzed the clinical and pathological significance of VM in 168 cases of laryngeal squamous cell carcinoma (LSCC) and found that VM occurred in LSCC, and LSCC with VM had increased potential for invasion and metastasis. Upile *et al*(8) showed that HN2b metastatic HNSCC cells lines have higher VM formation properties when compared with the HN2a primary tumor cell line, and endothelial growth factor antibodies discouraged VM formation.

Additionally, certain studies demonstrated that administration of angiogenesis inhibitors did not suppress the formation of VM, and even induced extracellular matrix-rich tubular network formation *in vitro*(19). Conceivably, VM may play a pivotal role as an alternative pathway for blood supply when the pattern of angiogenesis is inhibited.

Epithelial-mesenchymal transition (EMT) is a reversible dedifferentiation process that converts epithelial cancer cells into dedifferentiated cells with additional mesenchymal features. This process is characterized by the loss of epithelial traits and the acquisition of mesenchymal phenotypes (20–23). Activation of EMT triggers tumor cell invasion and metastasis to distant organs. Recently, EMT has been reported to contribute to the formation of VM, and the upregulation of EMT-associated transcription factors has been demonstrated in VM-forming tumor cells (24,25).

Normal tissues and tumors contain a small subset of cells, known as stem cells, with the capacity for self-renewal and the multipotency to differentiate into diverse committed lineages (26,27). Tumors are composed of diverse types of cells (28) and cancer stem cells (CSCs) are at the top of the hierarchical pyramid (26,29–31). Mounting evidence demonstrates that CSCs have the capacity for differentiation along tumor and endothelial lineages (32,33), as well as vascular smooth muscle-like cells (3). VM-engaging tumor cells show a significant expression of both endothelial and tumor phenotypes (14,34), and thus VM may represent the incomplete progress of CSC differentiation into endothelial lineages. Additionally, it has been observed that epithelial cancer cells may be endowed with the self-renewal stem cell phenotype via EMT (35,36). Therefore, in this review, we discuss the correlation between CSCs, EMT and VM formation.

## 2. Cancer stem cell (CSC) involvement in vasculogenic mimicry (VM) formation

CSCs, as defined by the American Association of Cancer Research, are a small subset of cells with the capability of self-renewal and differentiation into the heterogeneous lineages that constitute the tumor mass (26). In reality, this is only a functional definition. Due to the lack of specific markers, the so-called ‘CSCs’ obtained in almost all experiments are actually a mixture of real CSCs and progenitor cells. In that sense, it is also reasonable to call these cells tumor stem-like cells. Although there is controversy regarding the accurate definition of CSCs, increasing evidence supports the existence of CSCs and the validity of the CSC hypothesis (37). CSCs were first demonstrated in human acute myeloid leukemia (AML) when investigators found that the ability to initiate tumors by transplantation of AML cells into NOD/SCID mice was limited to a CD34^+^/CD38^−^ subpopulation of leukemic cells (38). CSCs have been further observed in several solid tumors, such as breast (28), brain (39,40), melanoma (41,42), prostate (43), ovarian (44,45) and pancreatic cancers (46), as well as HNSCC (47–53). In addition to the capability of tumor initiation, CSCs have also been implicated in tumor invasion and metastasis. In breast cancer, the CSCs sorted by a number of markers have a higher capability of invasion and metastasis. Balic *et al*(54) found that the majority of early disseminated cancer cells in bone marrow have a breast CSC phenotype (CD44^+^/CD24^−^). From a retrospective study of 109 patients with IBC, the patient prognosis and metastasis trends showed a significant correlation with aldehyde dehydrogenase 1 (ALDH1) expression, a specific marker of CSCs. Both *in vitro* and xenograft assays showed that invasion and metastasis in IBC are mediated by a cellular component that exhibits ALDH activity (55). In HNSCC cell lines, based on an invasive assay *in vitro* and injection of tumor cells into the tail vein of mice, Davis *et al*(56) found that CD44^+^ cells have an increased ability to invade through the basement membrane and to form lung metastases. In the peripheral blood of patients with HNSCC, a greater number of CD44^+^ tumor cells were also observed compared with that of the healthy control group (57). Song *et al*(58) demonstrated that side population (SP) cells in HNSCC were highly invasive, and the highly metastatic M3a2 and M4e HNSCC cell lines contained a greater number of SP cells in comparison with the 686LN parental HNSCC cell line that has low metastatic potential. It was deduced that SP cells may be a major driving force in head and neck tumor formation and metastasis. Goldie *et al*(59) reported that upregulation of FRMD4A, a human epidermal stem cell marker, occurs in primary human HNSCCs, where high expression levels correlate with increased risk of relapse. Additionally, FRMD4A silencing was shown to decrease the growth and metastasis of human squamous cell carcinoma xenografts in the skin and tongue.

Furthermore, Hermann *et al*(46) found that CD133^+^/CXCR4^+^ CSCs of pancreatic tumors are crucial in tumor metastasis and CD133^+^/CXCR4^−^ CSCs are associated with tumorigenesis. On the basis of this finding, CSCs are divided into two categories: Stationary CSCs, which are involved in tumorigenesis, and invasive CSCs, which are associated with the behaviors of invasion and metastasis. Moreover, resistance to conventional treatment has been considered as a problem in cancer therapy. At present, the exact mechanism of resistance is yet to be completely understood. Considering the slow proliferation rate (60), the higher resistance to the hypoxic environment (61) and cell death (62), and the role of the ABC family (such as ABCB1, ABCG2 and ABCB5) in pumping drugs out of the cell (63), CSCs are analogous to a reservoir of cells that survive the initial treatment and are responsible for tumor recurrence.

**Figure 1:**
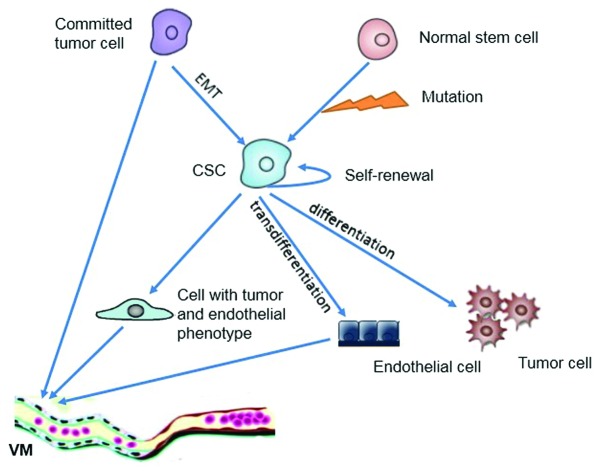
Schematic diagram representing the VM formation involved in CSCs and EMT. VM, vasculogenic mimicry; CSC, cancer stem cell; EMT, epithelial-mesenchymal transition.

With increasing knowledge of CSC phenotypes and functions, the evidence suggests that CSCs are involved in VM formation. In human breast cancer, by injecting human breast CSCs into SCID mice, Bussolati *et al*(64) found that a number of the intratumor vessels were of human origin, indicating the involvement of breast CSCs in vessel formation. In melanoma, there is evidence showing that the VM-forming tumor cells express phenotypes that are usually expressed in other cell types, such as endothelial or epithelial cells (11). This indicated that these cells may revert to an undifferentiated, stem-like phenotype (65). Recently, in glioblastoma, Ricci-Vitiani *et al*(32) and Wang *et al*(33) found that CD133^+^ glioblastoma stem-like cells are pluripotent and capable of differentiation along tumor and endothelial lineages (33,66), as well as mixed endothelial cell lineages, with co-expression of the tumor phenotype (32). On the analysis of tumor xenografts obtained by orthotopic and subcutaneous injection of human glioblastoma in immunocompromised mice, the authors observed that the vessels in the transplanted tumor were primarily composed of tumor cells with an aberrant endothelial phenotype. The findings indicate that these cells are derived from CSCs, and thus VM may represent an incomplete differentiation of cancer stem-like cells towards the endothelial lineage (Fig. 1). It has been reported that CD133^+^ and ABCB5^+^ subpopulations are colocalized in melanomas in perivascular niches that contain vascular endothelial (VE)^−^ cadherin^+^ melanoma cells, which have the ability to form VM (67). Frank *et al*(68) found that vascular endothelial growth factor 1 (VEGF-1) signaling plays an important role in this process and knockdown of VEGF receptor 1 blocked the development of ABCB5^+^ VM morphology. In oral squamous cell carcinoma, Dang and Ramos (69) observed that TRA-1-60^+^/β6^+^ tumor cells with CSC attributes are able to form vascular-like structures *in vivo*. However, based on the observation of the melanoma xenograft model, Zhang *et al*(70) found that VM was the dominant blood supply pattern in the early stage of tumor growth. During tumor growth progression, the level of VM decreased and the number of endothelial-dependent vessels increased. The authors proposed a three-stage blood supply pattern consisting of VM, mosaic vessels and endothelium-dependent vessels. This inverse change tendency between VM and endothelium-dependent vessels may be due to the persistent differentiation of CSCs to endothelial cells without tumor cell phenotypes.

## 3. Epithelial-mesenchymal transition (EMT) involvement in VM

EMT is a crucial process in cancer progression, providing cancer cells with the ability to escape from the primary site, invade stromal tissues and migrate to distant regions of the body. Epithelial cells undergoing EMT are characterized by downregulation of epithelial makers (such as cytokeratin), loss of cell polarity and intercellular adhesion molecules (for instance E-cadherin and occludin), which is concomitant with upregulation of mesenchymal markers (vimentin, N-cadherin and fibronectin) and acquisition of fibroblast-like morphology with cytoskeleton reorganization (20,22,71). The loss of E-cadherin and the gain of N-cadherin expression are known as cadherin switching, a major hallmark of EMT. Cadherin switching was observed in 30 out of 80 HNSCC cases and was closely correlated with histological differentiation, pattern of invasion and lymph node metastasis in HNSCC cases (72). According to analysis of HNSCC specimens and cell lines, Mandal *et al*(73) demonstrated a close correlation between EMT and aggressive tumor features, including penetrating invasive fronts, high-grade sarcomatoid transformation and lymph node metastasis. A variety of transcription factors such as Snai1 (Snail1) (74–76), Slug (Snail2) (77), Twist (78), SOX4 (79) and ZEB (80), and several signaling pathways, involving TGF-β, Wnt, Notch and Hedgehog, have been reported to play significant roles in the process of EMT (81–83). Snail and Slug repress E-cadherin transcription to degrade cell-to-cell adhesion by binding the E-box in the E-cadherin promoter, and inducing tumor cell migration (74,79,84). The transcription factor Twist, a master regulator of embryonic morphogenesis, contributes to metastasis of mammary carcinoma by promoting an EMT (78). Twist1 induced invasion and metastasis of hepatocellular carcinoma (HCC) via downregulation of E-cadherin and increased activity of matrix metalloproteinase (MMP), specifically MMP2 and MMP9 (85). In a spontaneous skin squamous cell carcinoma mouse model, Tsai *et al*(86) demonstrated that activation of Twist1 is sufficient to promote carcinoma cells to undergo EMT and disseminate into blood circulation. Aigner *et al*(87) identified that the transcription factor ZEB1 is able to induce the repression of certain polarity genes (Crumbs3, PATJ and HUGL2) to improve tumor cell invasion. Moreover, by virtue of repression of the miR-200 and miR-34a families, respectively, ZEB1 contributes to metastasis by maintenance of the dedifferentiation status and remodeling of cytoskeletal actin (88,89). Furthermore, there is evidence to suggest that these transcription factors function together, but not independently, in order to induce EMT (84,90).

Recently, evidence has shown that EMT is involved in the process of VM formation. In VM-positive colorectal carcinoma samples, Liu *et al*(24) found that expression of ZEB1 was upregulated. Downregulation of E-cadherin and upregulation of vimentin in the ZEB1-positive group were detected. Knockdown of ZEB1 resulted in a decrease in VM and the restoration of certain epithelial phenotypes, such as VE-cadherin and Flk-1. In HCC, the inhibition of Twist1 expression by the short hairpin RNA markedly reduced VM formation (25). Furthermore, the Bcl-2/Twist1 complex facilitates the nuclear transport of Twist1 and leads to transcriptional activation of a wide range of genes that may increase the tumor cell plasticity, metastasis and VM formation of hepatocellular carcinoma (91). Lirdprapamongkol *et al*(92) have reported that the poorly-differentiated HCC cell line, SK-Hep-1, with mesenchymal features (high invasiveness and expressing vimentin, with no E-cadherin) could form VM *in vitro*, while the well-differentiated cell line HepG2 did not form VM. These findings indicated that EMT is involved in VM formation.

## 4. CSCs are implicated in VM formation by the induction of EMT

Researchers have been engaged in discovering the origin of CSCs for a number of years. It is widely accepted that tumor formation is due to the multistep mutation of genomes. Considering the longer lifespan of stem cells, normal stem cells suffer from the accumulation of mutations over time. Thus, it is hypothesized that CSCs derive from normal stem cells with genetic mutations, and this has been demonstrated by independent investigators (93,94). In addition to this mechanism, mounting data suggest that differentiated tumor cells may reacquire stemness (95), particularly via EMT induction (35). The endowment of stem cell traits by EMT provided another source for the origin of CSCs. Biddle *et al*(96) classified the CSCs into two types, namely, non-EMT CSCs and EMT CSCs, based on EMT progression.

An increasing body of evidence shows that EMT is associated with the acquisition of CSC properties. In 2008, Mani *et al*(35) reported that the induction of EMT in immortalized human mammary epithelial cells results in the acquisition of mesenchymal traits, as well as the expression of stem cell markers. Stem-like cells isolated either from mouse or human mammary glands or mammary carcinomas express EMT markers (35,97). Morel *et al*(36) also indicated that cells possessing both stem and tumorigenic characteristics of ‘CSCs’ may be derived from human mammary epithelial cells following the activation of the Ras-MAPK pathway. The acquisition of these stem cell and tumorigenic characteristics is driven by EMT induction. Santisteban *et al*(98) found that breast tumor cells undergoing EMT induced by CD8^+^ T cells acquired certain characteristics of breast CSCs, including potent tumorigenicity, resistance to conventional treatment, and the ability to form spheroids. Fang *et al*(99) demonstrated that Twist2 is overexpressed in breast cancer cells. Ectopic overexpression of Twist2 results in the induction of EMT and increases the number of CD44^+^/CD24^−^ cells. Breast cancer cells exposed to TGF-β and TNF-α lead to the generation of breast cancer cells with stem-like characteristics by induction of EMT (100). In addition to breast cancer, Ryu *et al*(101) identified that the gastric CSC marker CD44 was significantly associated with the protein expression of Snail-1, ZEB-1 and E-cadherin. In colorectal cancer, EMT induced by brachyury increased the nanog expression and endowed the colorectal cells with stem cell attributes (102). In HNSCC, EMT conferring to stem cell phenotypes has also been observed. Xia *et al*(103) found that miR-200a regulates the acquisition of stem-like traits by the induction of EMT in nasopharyngeal carcinoma. Knockdown of miR-200a induced EMT progression and resulted in stem cell attributes, including an increasing proportion of SP, sphere formation capacity, *in vivo* tumorigenicity in nude mice and stem cell marker expression. Chen *et al*(104) revealed that HNSCC-ALDH1^+^ cells exhibit a high level of expression of Snail, and knockdown of Snail significantly decreased the expression of ALDH1. These data suggest that epithelial cells within tumors are able to convert into CSCs via EMT (Fig. 1). Moreover, Chen *et al*(105) demonstrated that upregulation of CD133 increased the phosphorylation of Src coupled with EMT transformation, and CD133/Src signaling is a regulatory switch resulting in EMT and stemness properties in HNSCC. This knowledge provides an improved understanding of the origin of CSCs and is a basis for novel cancer therapeutic strategies targeting EMT and CSCs.

VM allows tumor cells to express the endothelial phenotype and play a similar functional role to endothelial cells in forming blood vessel-like structures. In fact, both epithelial and mesenchymal markers have been observed in tumor cells engaged in VM formation (14,106,107). Therefore, in view of the crucial role of EMT in the acquisition of stemness, it is plausible that CSCs are implicated in VM formation by induction of EMT (Fig. 1). Signal transducers and activators of transcription 3 transcription factor plays a critical role in the development and progression of a variety of tumors, including HNSCC, by regulating cell proliferation, cell cycle progression, apoptosis, angiogenesis, immune evasion and EMT, and through effects in CSCs. Garnier *et al*(108) found that tissue factor overexpression accompanies features of cellular aggressiveness, such as markers of CSCs (CD133), EMT and expression of the angiogenic and prometastatic phenotype. Recently, Gill *et al* demonstrated that Snail promotes the induction of Flk1^+^ endothelial cells in an early subset of differentiating mouse embryonic stem cells, depending on fibroblast growth factor signaling as well as the repression of the miR-200 family (109). Hypoxia is one of the fundamental changes in the development and aggressiveness of a variety of solid tumors. It has been recognized to play critical roles in tumor invasion, metastasis, angiogenesis and chemo-radiation resistance. In addition to tumor angiogenesis, HIF-1a is closely associated with VM formation (4,110–112). Recently, Misra *et al*(113) found that hypoxia-exposure resulted in an upregulation of c-Myc and OCT3/4, and contributed to VM formation. Hypoxia was also recognized as an important regulator of CSCs and EMT through NF-κB, PI3K/Akt/mTOR, Notch, Wnt/β-catenin and Hedgehog signaling pathways (114,115). Thus, the hypoxia microenvironment may be important in VM formation through stemness maintenance and EMT induction.

## 5. Perspectives on cancer treatment

It is clear that tumors are able to grow to a size of ~1–2 mm^3^ depending on the diffusion of oxygen and nutrients (116). In order to break the metabolic restriction and meet the demands of growth, invasion and metastasis, tumors must form their own vessels to provide oxygen and nutrients, and remove metabolic waste. The microcirculation of tumors is heterogeneous, involving sprouting angiogenesis, vasculogenesis, co-opted vessels, mosaic vessels and VM. Angiogenesis was the first mode of vascularization to be discovered and has been extensively investigated. However, the success of anti-angiogenesis treatment remains limited (117). Keunen *et al*(118) found that anti-VEGF treatment with bevacizumab decreases the number of vessels and blood supply within the GBM xenograft, but it increases the invasion ability. Therefore, it is not sufficient to improve patient survival through anti-angiogenesis therapy alone.

In reality, the coexistence of angiogenesis and VM is common within aggressive tumors. Angiogenesis inhibitors have little or even no effect on VM (10,19) and VM may replace the effect of angiogenesis to provide the tumor with oxygen and nutrients. Moreover, Qu *et al*(119) reported that anti-angiogenesis therapy may even induce the formation of VM. Clearly, the combination of several treatments targeting angiogenesis and VM is required.

For quite some time, the survival rate of patients with aggressive tumors has remained at a low level, despite the administration of surgery, chemotherapy and radiotherapy. The existence of CSCs was thought to be an underlying cause. Although CSCs comprise only a small proportion of tumor cell populations, CSCs have high resistance to multiple chemotherapeutics and ionizing radiation. Remaining CSCs are able to induce recurrence following treatment with chemotherapy and radiotherapy. Furthermore, it has been demonstrated that CSCs are implicated in VM formation. In this context, CSCs have been considered as a promising treatment target in cancer patients with VM. It has been observed that tumors undergoing the process of EMT acquire resistance to chemotherapy (120). EMT is also involved in the acquisition of CSC properties (35,36,98), and EMT-inducing CSCs have been considered as an important origin of CSCs and another target of VM formation in cancer. A combination of targeting EMT and CSCs may be beneficial for anti-VM formation therapy, decreasing invasion and metastasis, and improving the survival rate of patients.
